# Coexistent Mitral Stenosis and left Circumflex Coronary Artery to left Atrial Fistula in a Patient with Severe Pulmonary Hypertension

**DOI:** 10.21470/1678-9741-2019-0297

**Published:** 2021

**Authors:** Yaming Shi, Yongzhong Zong

**Affiliations:** 1 Department of Cardiology, The Third People’s Hospital of Yancheng, Jiangsu, Yancheng, China; 2 Yancheng Third People's Hospital, Yancheng, China.

**Keywords:** Mitral Valve Stenosis, Atrial Fibrilation, Coronary Sinus, Fistula, Heart Atria

## Abstract

Coronary artery to left atrial fistula is rare in patients with mitral stenosis. We report an interesting case of a patient with concomitant mitral valve stenosis and coronary fistulae, originating from the left circumflex artery and drained into the left atrium with two terminal orifices.

## INTRODUCTION

Rheumatic heart disease is still common in developing countries. Mitral stenosis prevents left atrial emptying, increases left atrial and pulmonary venous pressure. The pulmonary arterioles may react with vasoconstriction, intimal hyperplasia, and medial hypertrophy, often resulting in pulmonary arterial hypertension^[1]^. A coronary artery fistula is defined as an abnormal communication between a normally originating coronary artery and another cardiac vascular structure. The incidence of coronary artery fistula is estimated to be less than 0.1% of patients undergoing diagnostic coronary angiography^[2]^. Coronary artery to left atrial fistula is rare in patients with mitral stenosis. We present an interesting case in which the fistulae originated from the left circumflex artery and drained into the left atrium with two terminal orifices.

## CASE REPORT

**Fig. 1:**
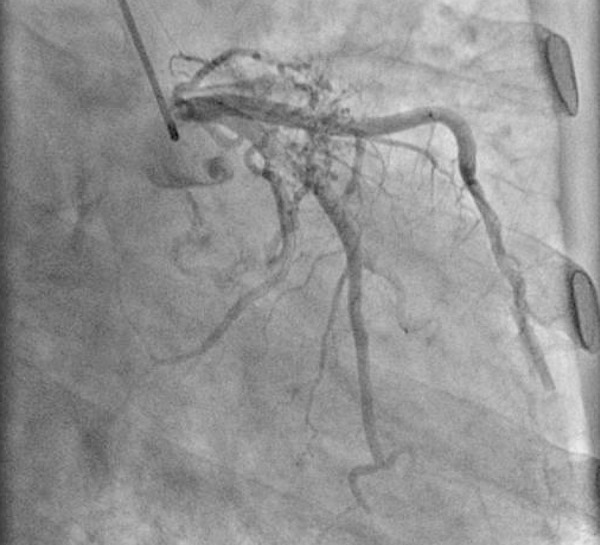
Right anterior oblique caudal view showing the fistulae originating from the left circumflex artery

**Fig. 2:**
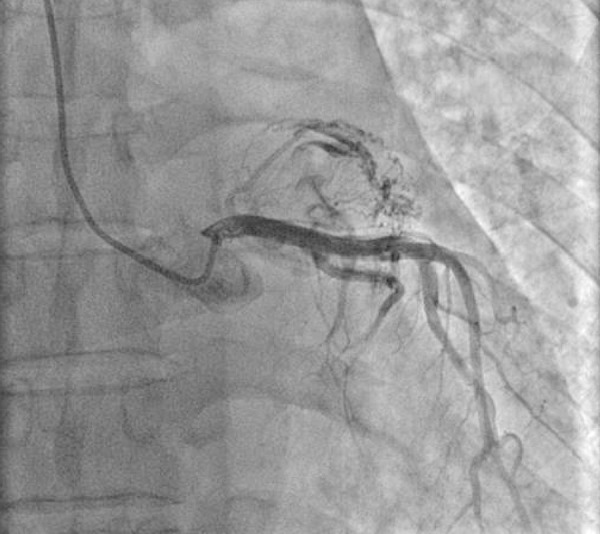
Antero-posterior oblique projection of left coronary angiogram revealed coronary artery fistulae drained into the left atrium with two terminal orifices.

**Fig. 3:**
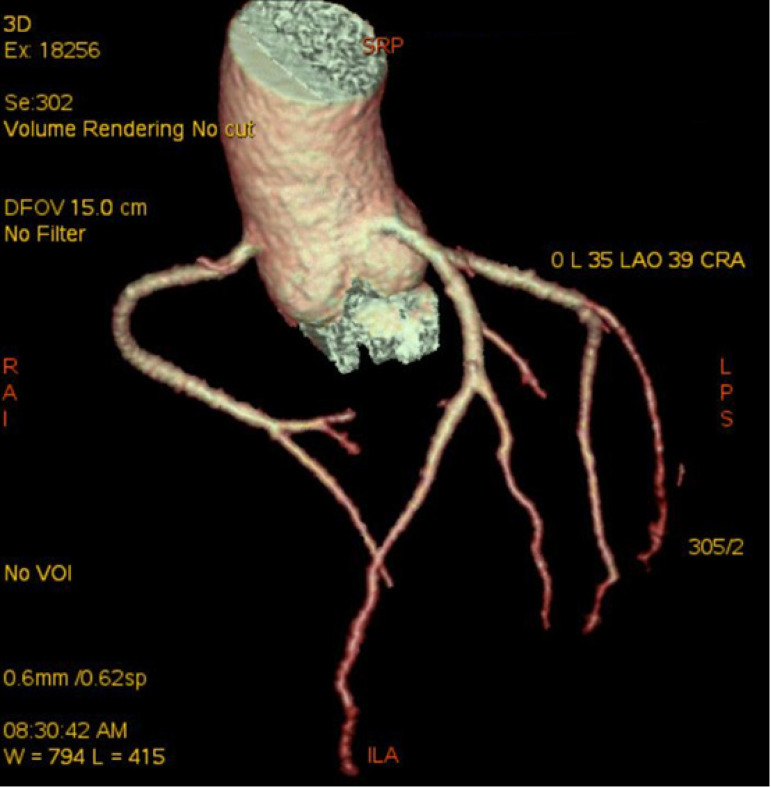
At 3 months postoperatively, the closure of the fistulae was confirmed by 128-slice computed tomography

A 57-year-old man was admitted to the cardiology clinic with a 20-year history of gradually increasing breathlessness on exertion and, in the previous 10 days, orthopnoea and paroxysmal nocturnal dyspnoea. Physical examination found irregular pulse with diastolic rumbling murmur. The electrocardiogram revealed atrial fibrillation with a ventricular rate of 120 beats/min and accompanying T wave abnormalities and minimal ST depression in inferior derivations. Chest radiograph revealed double shadow on the right cardiac silhouette and prominent pulmonary trunk with increased vascular markings. Transthoracic echocardiography revealed a mitral stenosis with a mitral area of 0.7 cm^2^, ejection fraction of 68%, and normal segmental wall motion, mild aortic regurgitation, severe pulmonary hypertension and moderate left atrial enlargement. Severe pulmonary hypertension was confirmed by Doppler (pulmonary arterial pressure = 106.8 mmHg). The patient received 20 mg furosemide, 40 mg spironolactone, 0.125 mg digoxin and low molecular weight heparin by subcutaneous injection. With the relief of dyspnea, coronary angiography was performed for preoperative evaluation of mitral valve replacement. Right anterior oblique caudal view showed coronary artery fistulae between the left circumflex artery and the left atrium. One large fistula originated from the first obtuse marginal branch and the second obtuse marginal branch, and another fistula originated from the third branch of the second obtuse marginal branch (Figure 1). Antero-posterior oblique projection of the left coronary angiogram revealed the coronary artery fistulae drained into the left atrium with two terminal orifices (Figure 2 and Video 1). The fistulae were hemodynamically significant, and closure was indicated. In the operation, utilizing cardiopulmonary bypass, the mitral valve was replaced by a 27-mm ATS Open Pivot Bileaflet Heart Valve (ATS Medical Inc., Minneapolis, MN). At the time of mitral valve replacement, the fistulae were successfully ligated through not only left atrium but also the left circumflex coronaries’ side. Postoperatively, the patient made an uncomplicated recovery. At 3 months postoperatively, the transthoracic echocardiography was performed, which revealed pulmonary arterial pressure of 62 mmHg, and the closure of the fistulae was confirmed by 128-slice computed tomography (Figure 3).

**Video 1:**
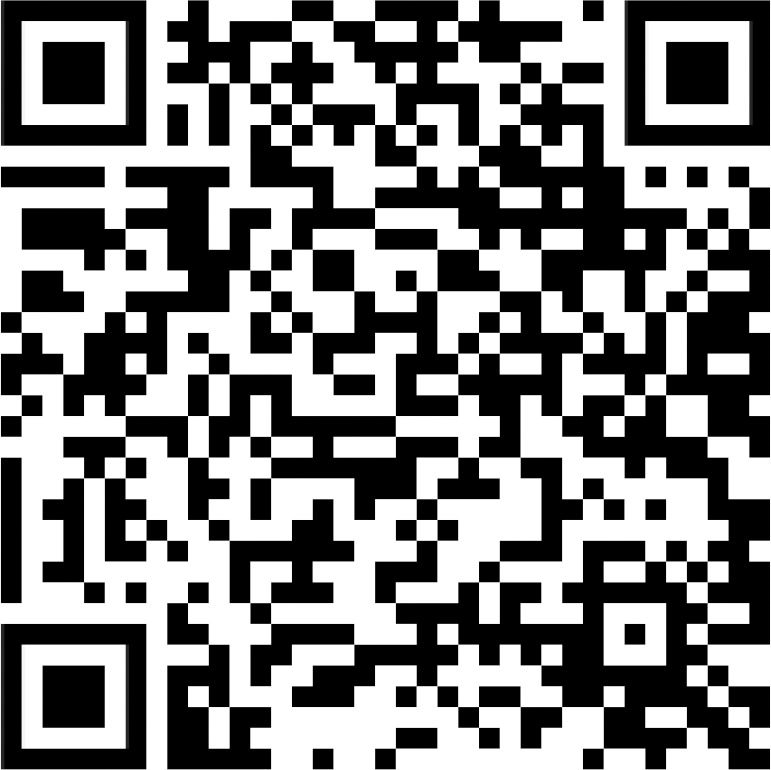

Coronary artery fistula is a rare anomaly connecting coronary arteries to cardiac chambers or great vessels, which are rarely detected during routine angiographic evaluation. The frequency of congenital coronary fistulas is reported at approximately 0.1%^[2]^. Mitral isthmus ablation, which is an important component of catheter ablation for persistent atrial fibrillation and mitral isthmus-dependent flutters, become one of the reasons to cause a fistula between the left circumflex artery and the left atrium^[3]^. The main sites of origin are the right coronary artery (55%), the left coronary artery system (35%), and both coronary arteries (5%). The main termination sites are right ventricle (40%), right atrium (26%), and pulmonary arteries (17%). Less frequently, they may drain into the superior vena cava or coronary sinus and less frequently into the left atrium or left ventricle^[2,4]^. Although a similar fistula is reported with one entrance draining into the left atrium in the literature^[5]^, to our best knowledge, this is the first case of the circumflex to left atrium fistulae with two terminal orifices.

Although asymptomatic in the vast majority, coronary artery fistula may cause chronic myocardial ischemia and angina, congestive heart failure, myocardial infarction, pulmonary hypertension, rhythm disturbances, subacute bacterial endocarditis, thromboembolism, rarely aneurysmal segment rupture, and sudden death^[6]^. Small fistula usually does not cause hemodynamic impairment. However, high volume shunts via left circumflex coronary artery to the left atrium may result in increasing volume load of the left atrium and pulmonary arterial hypertension. In our reported cases, coronary fistulae and mitral stenosis appeared together may just be a coincidence, and pulmonary arterial hypertension may be due to the chronic left atrial volume overload caused by congenital coronary-left atrial fistulae and mitral valve stenosis. The best way to manage coronary cameral fistulae is not well known due to the rarity of the condition. Surgical repair of coronary artery fistulae is safe and effective, with low risks and favorable late outcomes^[7]^, as described in this case.

**Table:** 

Authors' roles & responsibilities	
YS	Substantial contributions to the conception or design of the work; or the acquisition, analysis, or interpretation of data for the work; drafting the work or revising it critically for important intellectual content; final approval of the version to be published
YZ	Substantial contributions to the conception or design of the work; or the acquisition, analysis, or interpretation of data for the work; drafting the work or revising it critically for important intellectual content; final approval of the version to be published
